# Identifying indicators and evaluation steps with suggestions for improving trauma care in Iran: experts’ perspective

**DOI:** 10.5249/jivr.vo113i2.1589

**Published:** 2021-07

**Authors:** Yalda Mousazadeh, Ali Janati, Mahboub Pouraghaei, Homayoun Sadeghi Bazargani

**Affiliations:** ^a^ Department of Health Policy and Management, Iranian Center of Excellence in Health Management, School of Management and Medical Informatics, Tabriz University of Medical Sciences, Tabriz, Iran.; ^*b*^ Iranian Center of Excellence in Health Management, School of Management and Medical Informatics, Tabriz University of Medical Sciences, Tabriz, Iran.; ^*c*^ Emergency Medicine Research Team, School of Medicine, Tabriz University of Medical Sciences, Tabriz, Iran.; ^*d*^ Road Traffic Injury Research Center, Tabriz University of Medical Sciences, Tabriz, Iran.

**Keywords:** Indicator, Evaluation, Trauma center, Trauma care, Iran

## Abstract

**Background:**

Trauma is one of the major causes of mortality across the world. Trauma patients have critical status and need timely, adequate, and organized care. The different consequences of trauma care among service centers around the world and even within a country revealed the need for careful evaluation. This study was designed and executed to collect experts’ opinions on the evaluation steps, related indicators, and improvement strategies in trauma care.

**Methods:**

This qualitative study was based on a conventional content analysis approach. 2 focus group discussions (FGD) with 6 participants per FGD and 16 face-to-face in-depth interviews were conducted to collect the required information (from September 2018 to early 2019). Participants were selected through the purposive sampling method. The experts’ viewpoints were classified by the main and sub themes.

**Results:**

Four basic themes extracted from the interviews and focus group discussions including, trauma care importance (sub-themes: the involved individuals’ being young and productive and the effectiveness of trauma care); trauma care indicators (sub-themes: pre-hospital indicators, in-hospital indicators, and post-hospital indicators); stages of trauma care evaluation (sub-themes: evaluation prerequisites, finalization of indicators before the evaluation, determining evaluation time scope, determining evaluation dimensions, external and internal evaluation and use of evaluation results); trauma care improvement (sub-themes: balancing workload in trauma centers, enhancement of information system, considering extra-organizational dimensions in trauma care and empowerment of trauma care providers).

**Conclusions:**

According to experts’ viewpoints, trauma is a very important issue, because it involves young people. They believed that having indicators covering all aspects of care assist health managers and policymakers to understand under-standard performance. These indicators should be used in the form of a specific evaluation program and related to Iran context. Besides, reforming macro policies, planning, development of infrastructures, and education was some recommendations of experts to improve trauma care.

## Introduction

Trauma is an inevitable cause of death across the world.^1^ Traumatic injury cause higher mortality rates compared to Acquired Immune Deficiency Syndrome (AIDS), malaria, and tuberculosis.^2^ The number of deaths caused by trauma is estimated to be 5.8 million per year.^3^ The majority of dead people are below 45 years of age.^4^ Injured people are patients with the high financial burdens.^5^ Such injuries lead to 52 million Disability-Adjusted Life Years (DALYs) and contribute to 15% of the global burden of disease.^6^ In Iran, trauma is considered to be an important issue. It is the second cause of Iranian’s death and the first cause of death among youth. Further, it is the most important cause of DALY.^7^ 28% of DALYs were attributed to trauma in Iran.^8^ According to estimations, the implementation of plans for improving care services to trauma patients will save 1,730,000 to 1,964,000 people annually in low and middle-income countries (LMIC).^2^

Trauma management needs quick, pre-determined, and cost-effective performance for the injured people.^9-10^ Unfortunately, half of trauma patients do not receive adequate cares in studies. Medical errors are common and preventable deaths are reported in hospitals.^10^ There are some evidence indicating that clinical care level is below standard and trauma outcomes are different in trauma centers.^4^ Moreover, health care authorities have declared that due to the remarkable increase in health care costs, it is necessary to collect data on health care performance.^10^ According to evidence, paying attention to data associated with the cause of different performances and taking necessary measures in this regard improves the quality and efficiency of care providing centers.^11,12,13^ In fact, health care services have low chance of improvement unless they are evaluated correctly.^9^ Evidence of service quality improvement has been cited in some studies. Niemeijer et al. improved the discharge procedure of patients by the six-sigma model at the Trauma Nursing Department (TND). The average length of stay (LOS) was 10.4 days at the beginning of the project. After the implementation of the improvements, the average LOS was 8.5 days.^14^ Two studies in Thailand assessed the rates of preventable deaths before and after applying trauma filters related to trauma care. From 1994 to 2000, there was a major reduction in preventable deaths from 3.2% to 1.3%.^15-16^

For the first time, the trauma Committee of the American College of Physicians and Surgeons formulated some indicators for quality measurement. Initially, these indicators contained 12 auditing filters. Later, the number of them increased and they were separated into general and special indicators.^17^ Although using evaluation methods of developed countries may be beneficial, but high-income countries use indicators that require a high level of resources. For example, adequate and trained personnel, timely access to advanced diagnostics, etc., which are difficult to use in low- and middle-income countries. It seems necessary that they should be localized per conditions and databases of developing countries.^18^ In Iran, the Ministry of Health and Medical Education has defined five criteria as the key performance indicators of hospital emergency services (not trauma services only) to measure and evaluate the performance of emergency centers. The indicators are the percentage of patients’ deposition within 6 hours and deposition within 12 hours, unsuccessful CPR (Cardio Pulmonary Resuscitation), discharge against medical advice and mean triage time.^19^ Nevertheless, no pre-defined and specific indicator and evaluation method were found about trauma care in Iran. It is said that resorting to specialists’ opinions provides some principles for trauma care and prepares it to change.^20^ Therefore, this study was designed and implemented to collect experts’ opinions on the evaluation method, related indicators, and improvement strategies in trauma care.

## Materials and Methods

This was a qualitative study with a conventional content analysis approach. The reason for using the qualitative method was the ability of these studies in drawing out the participants’ experiences, knowledge, and unraveled information.^21^ The study was conducted from September 2018 to early 2019. It was a part of a broader project titled “Developing a hospital performance assessment model for patients management with traffic injures”. The conventional content analysis approach is used to interpret implications derived from the content of text data.^22^ Focus group discussions and semi-structured in-depth interviews were used to collect the views of 28 trauma care experts about evaluation steps, related indicators, and improvement strategies in trauma care.

**Focus group discussion**

Two focus group discussions (FGD), each of which lasted for 60 to 90 minutes, were held with six participants per FGD. The participants were selected through the purposive sampling method who have the largest and richest information.^23^ It continued until data saturation, which means reaching a point where the researchers feel the new information does not come with the arrival of new people. Participants were physicians, who were involved in managing and providing care to trauma patients, or researchers, who had researched in trauma care and its evaluation. All participants had at least 5 years of work experience and the desire to participate in the study. Each focus group discussion was administered by three persons: the coordinator, the scriber, and the observer. At the beginning of each session, the researchers were provided with an adequate explanation about the objectives and method of FGD. Also, all of the participants were assured that the information provided by them would be completely confidential. During the sessions, the questions were asked by the coordinator, transcribed by the scriber, and recorded by a voice recorder. Besides, the observer tried to consider all the participants and to further engage those who rarely contributed to the discussions. 

**Interview**

16 interviews were used for data collection. It should be noted that the participants in the interview were those who did not participate in the group discussion due to their busy schedules. Each interview was in exclusive one-to-one. Before each interview, an interview guideline was provided by literature review and experts’ views. Some interview questions included: 

1. What is your opinion about the importance of trauma care? 

2. What are the indicators for the evaluation of trauma care? 

3. What are the characteristics of the evaluation? 

4. How to evaluate? When? By whom? How to use the results?

5. What are the barriers to evaluating and collecting information about indicators? 

6. Does the evaluation through the defined indicators affect the quality of the trauma care? 

7. What are your suggestions for improving trauma care? 

The selection of the experts was based on purposive sampling, which was followed by snowball sampling^24^ and continued until information saturation. The criterion for selecting these people was to have the most information about the subject of the study (similar to FGD). Phone coordination was performed before each interview. Interviews were face-to-face and each interview lasted for 45 to 60 minutes. Before each interview, a datasheet, including a summary of the study objectives, the interviewer’s information, interview date, and questions were sent to the participants. The location of interviews was selected by the interviewees’ tendency and, they were done in the participants’ workplace. All of the interviews were recorded by a voice recorder and were noted down by the interviewer. 

**Data analysis**

All the recorded data were transcribed word by word and then they were compared with the interviewer’s notes during the interview. Next, they were analyzed through content analysis. Data analysis and coding processes were as follows: familiarizing with the data text (reading the transcripts several time-data immersion), identifying and extracting the basic codes (identifying and extracting more relevant data with the primary codes), identifying themes (placing the initial extracted codes in related classes and themes), reviewing and completing the identified themes, naming and defining themes, re-coding and renaming some classes and themes, and ensuring the reliability of the codes.^21^

The procedure was as follows, one of the researchers separately coded each interview and focus group discussion in a pre-prepared form, and a list of these codes was compiled. Two members of the research team studied the codes independently and put their comments and their changes. Then, the research team discussed the main subjects during some meetings. At the end of the meetings, they reached a consensus about the study codes and themes. Finally, the themes were categorized by researchers. Next, all sub-themes were based on a logical and thematic relationship with each other in the main categories, which were the main themes. 

**Validity and reliability of data**

To promote the accuracy and reliability of data, they were reviewed by the participants. In other words, a summary of the transcribed data was returned to the participants to let them approve data accuracy.^25^ Then, their comments were applied. At this point, some of the researchers' misconceptions about experts' views were corrected. For Credibility and Confirmability, the researchers were involved a long time with the data. In addition, to achieve triangulation the data were coded and the themes were determined by the consensus of the research team.^25^ Moreover, for the Transferability, the expert review, purposeful sampling, and heterogeneous sampling were used.^22^

## Results

**Participant profile**

The participants included physicians working in two public trauma centers, university professors, researchers of trauma care, and evaluation experts of health services. The majority (88.88%) of the participants were male. More than 80 percent of participants had more than 10 years of work experience. The age range of all participants was 30 to 60 years. The demographic information of the participants is shown in Table 1.

**Table 1:** Demographic information of the participants.

Variable		Number	Percent
**Sex**	Female	4	11.12
	Male	24	88.88
**Age (year)**	30-40	6	21.43
	41-50	15	53.57
	51-60	7	25
**Specialty**	Emergency medicine	4	14.28
	Orthopedist	3	10.71
	Anesthesiologist	1	3.58
	General surgeon	2	7.14
	Neurosurgeon	2	7.14
	Internist	1	3.58
	General physician	4	14.28
	Nursing	6	21.43
	Epidemiologist	1	3.58
	Health services management	3	10.7
	Medical records	1	3.58
**Work experience (year)**	5-10	5	17.86
	11-20	12	42.86
	21-30	11	39.28

The experts addressed four main topics including trauma care importance, trauma care indicators, stages of trauma care evaluation, and trauma care improvement. All themes and sub-themes are presented in Table 2.

**Table 2:** Main and subthemes of study.

Item	Main themes	Sub themes
1	Trauma care importance	The involved Individuals’ being young and productive
		The effectiveness of trauma care
2	Trauma care indicators	Pre-hospital indicators
		In-hospital indicators
		Post-hospital indicators
3	Stages of trauma care evaluation	Evaluation prerequisites
		Finalization of indicators before the evaluation
		Determining evaluation time scope
		Determining evaluation dimensions
		External and internal evaluation
		Use of evaluation results
4	Trauma care improvement	Balancing workload in trauma centers
		Enhancement of information system
		Considering extra-organizational dimensions in trauma care
		Empowerment of trauma care providers

**1. Trauma care importance**

According to the experts’ perspective, owing to the significance of trauma care, function and evaluation of trauma care performance can be considered from two perspectives:

***A. The involved individuals’ being young and productive***

All participants pointed out the most trauma-affected people are young. Indeed, they are at production and activity age. Usually, the age of them is under 45 years. On the other hand, most of the injured people are among those who have been remarkably invested in terms of education and so on. 

*“… Unfortunately, trauma is one of the major causes of mortality especially in the first 4 decades of life…” (P20).*

***B. Effectiveness of trauma care***

Experts believed that most trauma patients are patients with acceptable prognosis. Because they are young (for example patients with car accident injuries). They may not have another chronic disease that exacerbates their status. Therefore, providing a timely care improves therapy, makes it effective. Conversely, they may seriously damage, if they do not receive timely diagnosis. 

*“…Fracture, is the simplest trauma that may occur among young people in car accidents. However, it can be treated quickly by using a simple care service. However, if the care is not accurate, this fracture may turn into an open fracture. In this case, the edge of the bone can cut through and injure the skin and result in strange events ranging from infection to embolus...” (P11).*

**2. Trauma care indicators**

The experts confirmed that indicators of different phases of trauma should be determined to identify and evaluate whether trauma care services are provided acceptably. Then, by comparing current procedures with relevant standards, judgments may be made on current trauma services. These indicators with some participants’ direct quotes are summarized in Table 3

**Table 3:** Trauma care indicators from the participants’ perspective.

Sub-themes	Indicators	Direct quotations of participants
**Pre-hospital indicators**	• Time interval of patient transportation (scene and hospital)	“…Many patients’ status at this pre-hospital stage can affect their next condition in hospital…” (P1)
	• Appropriate immobilization	“...Such indicators highlight the importance of the completeness of assessing and stabilization of patients in pre-hospital phase, for example, immobilization, bleeding and, breathing…” (P6)
	• Appropriate serum therapy	“…Suppose, for example, two patients, one with shoulder fracture and the other with a femur fracture, have been transported to the hospital. Relying on his/her previous knowledge, the ambulance technician in charge thinks that the priority is with the patient with a femur fracture. But, if the technician let the nurse know that the one with shoulder fracture suffers arrhythmia too, the nurse will surely give priority to this patient…” (P1)
	• Patients’ airways control	
	• Bleeding control	
	• Knowledge of ambulance technician	
	• Adequacy of ambulance equipment	
	• Completeness of pre-hospital reports	
**In-hospital indicators**	• Availability of clinical guidelines and act accordingly	“…Hospital care service indicators should be derived from clinical guidelines. clinical guidelines are algorithms used to initiate patient evaluation, administer the treatment, complete treatment process, define care key points, evaluate treatment trend, and measure the patient's recovery rate…”(P6)
	• Appropriate triage	“…Cooperation and consultation of specialists in the form of trauma team is of high importance. Suppose that a patient needs brain surgery and at the same time suffers internal problems too. In this case, if specialists intervene under the standards of their field of study, the procedure will damage the patient. In this case, the specialists’ team should assess priorities and determine which problem should be prioritized…” (P2)
	• Waiting time for doctor’s visit	“….Patients with high triage levels are in critical condition. For example, patients with severe fractures and low consciousness have the critical conditions. Patients with abdominal pressure require laparoscopy. The time it takes to transport such patients to surgery rooms is a vital indicator…” (P6)
	• Active trauma team	“…After GCS is determined, particular measures should be taken. Generally, when this indicator falls below 8, intubation is recommended…”(P1)
	• Waiting time for receiving Para clinical services	“…GAP as a tool for identifying preventable mortality classifies patients into three classes: mild, moderate, and severe. The percentage of death rate in each group is definite. Given some factors such as age, blood pressure, and GCS, The GAP score can be calculated for the hospital under study. Then, the obtained score for each group of the patients can be compared with global value…” (P20)
	• Injury severity and survival likelihood	“…The physical space of emergency centers should be designed ergonomically, that is the equipment with similar applications should be laid out in the same direction…”(P6)
	• Disposition time including discharge time, hospitalization inwards, transportation time to another center, time for transporting patients to surgery rooms	‘…Injury severity and survival likelihood represent preventable deaths and are used to compare the quality of service …” (P25)
	• GCS control	“…Providing timely and accurate medical, and nursing services are considered as the determinants of care operations…”(P16)
	• Adequacy of equipment, human resource, and physical space	“…Input is always the first-rank priority and to provide appropriate services minimal degrees should be determined …” (P15)
	• Occurrence of unwilling cases	“…Death in critical care hospitalization is frequently used as the main outcome indicator in injury research…” (P18)
	• Hospital-acquired infections	“…Specialists argued that a group of indicators may be important at the national level where they are collected throughout the country. Therefore, adopting these indicators in trauma cares may be beneficial for example CPR, Occurrence of unwilling cases, and Hospital-acquired infections…’(P11)
	• Registering time and information of patients	
	• Respiratory cares	
	• Bed sore cares	
	• Post-surgery cares	
	• Creating appropriate feeding ways	
	• CPR of trauma patients	
	• Hospital mortality	
	• Preventable mortality	
	• Injury severity	
**Post hospital indicators**	• Transferring to rehabilitation facilities	“…Many patients become disabled due to trauma and they should be continuously checked by physiotherapists and at times be referred to rehabilitation centers…” (P9)
	• Re visit in clinic and physiotherapy center	“…Assessing the individual’s quality of life, returning him/his to pre-vious normal life and obtaining acceptable functioning status are other dimensions of post-hospital assessments…” (P10)
	• Multiple hospital visits	“…GOS categorizes a patient within a range varying from recovery to death. The subcategories of this criterion are health, mild disability, moderate disability, vegetative state, and death…”. (P5)
	• Evaluation of performance using GOS or other tools	
	• Interventions of the social worker on trauma patients (including referring to patients’ home and assessment of mental state)	

GCS: Glasgow Coma Scale, GOS: Glasgow Outcome Scale, CPR: Cardio-Pulmonary Resuscitation, GAP: Glasgow Coma Scale, Age, Sys-tolic Blood Pressure

**3. Stages of trauma care evaluation**

The experts claimed that after the mentioned indicators are determined, it is possible to measure trauma care services under defined stages. These stages included evaluation prerequisites, finalization of indicators before the evaluation, determining evaluation time scope, determining evaluation dimensions, external and internal evaluation, and use of evaluation results.

***A. Evaluation prerequisites***

All evaluations need a competent leader based on experts’ perspectives. Also, before evaluations, all service providers should be informed of the evaluation procedure and learn how to use the obtained results and yield its results. The experts pointed to trauma care evaluations should be made by the level of facilities and the nature of activities. They believed that data are the key elements of evaluations. Selecting a certain person for collecting data for evaluation is of high importance. 

*“….The process of trauma care evaluation should consider different levels of trauma centers and their capabilities to enable the accurate benchmarking of the centers…” (P3)*

***B. Finalizing indicators before evaluation***

The experts argued that the consensus should be achieved as to the studied indicators. Indeed, the indicators, themselves, need to be monitored. This monitoring includes investigating if the indicators can be used and are necessary at different times. According to the experts’ view, the indicators should be evidence-based and should be in connection with outcomes. Also, it is important to data can be collected based on available money and facilities.

*“…After revising indicators, we may realize that some have no relationship with pleasing/non-pleasing outcomes and should be discarded…” (P4)*

***C. Evaluation time scope***

The experts argued that an indicator that is most linked to mortality, should be studied within shorter intervals. All of the participants mentioned to indicators should be prioritized. The priority belongs to the indicators that have a direct relationship with death. Depending on indicators, evaluations may be practiced on a daily, weekly, monthly, or annual basis. Therefore, consensus should be achieved about evaluation time scope before initializing evaluation.

*“…Doctor’s attendance to patients’ bed may be checked daily. Also, mortality rate and the quality of services may be evaluated on a monthly and weekly basis, respectively... ” (P24)*

*“… For a patient with respiratory distress, who needs to be emergently intubated, the time between doctor’s visit and making decision on intubation should be checked daily or, even, per case. Some indicators such as hospital-acquired infections with lower incidence can be evaluated once every six months, or even, annually…” (P7)*

***A. Dimensions of evaluation***

The experts believed that evaluations should have broad dimensions and any given problem should be studied from different perspectives. Indicators may be defined at the first step, and then, processes or operations comparisons may be made with ideal values. Assessment of awareness, knowledge, attitude, and skill of service providers is another part of the evaluation to realize whether staffs know how to perform their tasks and how they should perform them ideally. The assessment and use of service receivers’ opinions are important too. Therefore, it should certainly be evaluated in the framework of patient satisfaction measurement. 

*“…Evaluation is effective when it is comprehensive. It should include reviewing process adequacy, staff skills, and patient satisfaction...”(P8)*

***B. External and internal evaluation***

The experts asserted that external evaluations are generally performed by auditor organizations, such as accreditation organizations. Principally, it is better to select out-organization auditors because they are not a stakeholder of the studied system. Also, some services need to be monitored the therapy process for example intubating. For on-job evaluations, it is better to use in-organization employees, who are familiar with the processes of the provided services but do not individually benefit from the procedure. This study also demonstrates that modern trauma care monitoring techniques are currently being used. For example, the interventions on trauma patients are recorded by cameras and then, judgments are made concerning the accuracy and adequacy of the interventions. 

*“…Whenever evaluation and execution are merged, a potential corruption is generated because personal interests may be taken into account in the evaluation process. However, some processes are unique and rare, and sometimes people within the organization must be used for evaluation at the time of the event…” (P12)*

***C. Use of evaluation results***

All of the participants believed that it is better to investigate evaluation results within hospital committees to follow up relevant feedbacks through relevant clinical groups. Then, they should be submitted to relevant authorities. Eventually, both positive and negative results along with promotion outcomes should be made public through media. 

*“…It is better to investigate 5 cases of the incidences along with patients’ records and relevant documents in different committees to discover the root causes of such events…” (P14)*

**4. Improvement of trauma care**

**Figure 1:**
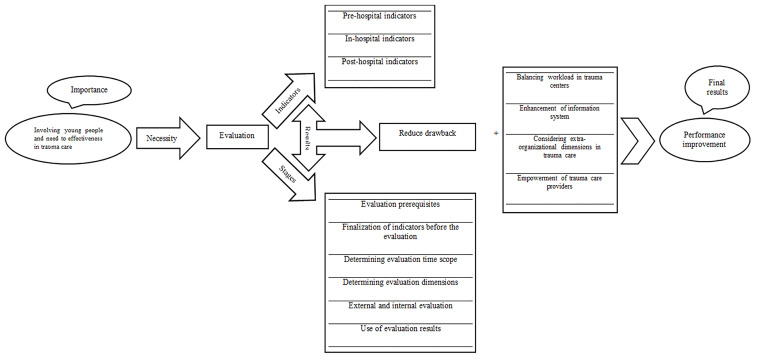
Stages evaluation and performance improvement in trauma care

This study highlighted that in addition to the assessment and elimination of weaknesses and drawbacks, some interventions improve trauma care. These interventions included balancing workload in trauma centers, enhancement of information system, considering extra-organizational dimensions in trauma care, and empowerment of trauma care providers (Figure 1).

***A. Balancing workload of trauma centers***

The experts declared that although the shortage of trauma care centers is a challenge in providing trauma care services, the most important problem in Iran is the adequacy of binding to referring system which, in turn, leads to balanced workload and appropriate services. Considering trauma centers in terms of covered population creates this balance. The adequate number of centers among provinces and regions must be observed. Even the referrals should be considered among trauma centers in an area by specialization.

*“…If trauma poles are defined in all provinces, the workload will be distributed between the centers…” (P13)*

***B. Enhancement of information systems***

Experts insisted on the improvement of trauma care requires the improvement of the information system. Data are the improvement resources of any system. Data collection seems time-consuming; however, if proper data are collected, this will facilitate the process of decision-making, and consequently will promote quality. 

*“…To show the importance of data, data collectors should be appreciated. Also, the outcomes of data utilization and their role in the improvement of the process can be publicized…” (P26)(P27)*

The experts stated that building a trauma registry system could aid the improvement of trauma care. If all data of trauma patients are fed into the system and then analyzed, in addition to helping make judgments on the quality of services, they will assist doctors to make proper decisions in the future. 

***C. Extra-organizational dimensions in trauma care***

According to the experts’ views, trauma care improvement depends on the reduction of faults and human errors in communities. On the other hand, it at times turns into a social and extra-organizational problem. Sometimes, the condition of the roads or the defect in the produced cars is involved in traffic accidents leading to trauma (for example). Trauma indicators are broad social indicators where even an education-oriented behavior may affect trauma incidence. It needs to develop people's perception and cooperation of many organizations in society. 

*“…In-car accident-induced traumas, the promotion of traffic culture, and road and car safety are likely the necessary actions to be taken…” (P3)*

***D. Empowerment of trauma care providers***

Trauma care is a specialty field based on experts’ perspectives. The promotion of trauma cares needs fully specialized staff in the medicine and nursing sectors. Providing therapies and cares following relevant protocols, and educating employees based on such protocols will always improve trauma care. Particular training is necessary for trauma care providers in the cause of its multidisciplinary nature. Experts argued that wherever the subject of education is introduced, it results in changes. 

*“…There has been always this wrong view that unsuccessful and sloth nurses are transferred to emergency centers to continue their job there whereas trauma cares demand specialized and routine services requiring trained nurses, not temporary personnel…”(P23)*

This study confirmed that consistency of evaluations is a success factor by itself and it converts decisions to actions. Monitoring improves performance almost by 20%. There is a proper infrastructure due to the availability of some programs such as quality improvement, clinical governance, and evaluation departments, the development of which enhances individuals’ knowledge on the issues of quality and patients’ safety.

## Discussion

The quality improvement methods have been used in developed countries for years ago in the cause of differences in patient care. These methods and tools may not be compatible with the conditions of developing countries. The lack of infrastructure and evaluation programs indicates that there is a need to localize tools and methods, and expert opinions can be helpful in this regard. In this study, experts described indicators including pre-hospital, in-hospital and, post-hospital according to the Iran health system. They believed an evaluation program assist to understand under-standard performance. Experts also described the steps of trauma care evaluation and suggestions for better service delivery. Modification of referral system, training, enhancement of information system and considering extra-organizational issues included experts’ suggestions for improvement.

Experts confirmed that many trauma patients are young and likely to have no illness or problem beforehand. Therefore, if these patients are properly managed and receive organized medical, diagnostic, and rehabilitation services, they will recover and return to life.^25^ Sometimes, the best treatment is not provided to trauma patients. According to the study of Ahmadinejad et al. on trauma patients admitted to Intensive Care Unit (ICU), 22.9% of discharged patients and 24.3% of dead patients showed at least a positive bacterial test during the hospitalization period.^26^ Mousazadeh et al. reported many mortalities due to traffic accidents occurred in the low risk group so, it may be related to the quality of care services.^27^

Several indicators were defined in different phases of care from the perspective of the participants in the present study. They are essential components of performance improvement in studies. There is no consensus on the indicators employed. Nevertheless, the use of indicators is common in trauma centers.^28^ Indicators vary in developing and developed countries. To provide appropriate recommendations to the needs of low- and middle-income countries, the Essential Trauma Care Project was established in collaboration with the WHO and participants from developing countries. 11 core essential trauma care services were the results of this project that can reasonably be provided to every injured person in every country.^3^ In other efforts, Mousazadeh et al. identified 50 trauma care indicators that can be used to assess and improve performance and compare trauma centers in Iran and developing countries.^29^

In this study, the experts presented the indicators according to Iran context that their measurement seemed necessary. Such efforts have been seen in similar studies. In the study carried out by Santana et al., 31 indicators were identified in the areas of structure, process, and outcome to evaluate the safety, effectiveness, efficiency, time interval, justice-orientation and, patient-centered after literature review and holding four Delphi rounds. These indicators were used in the United States, Australia, Canada, and New Zealand to assess trauma centers.^10^ In a research study conducted by Morrie et al., the indicators were developed by expert consensus, and then they were collected data from 59 trauma centers of the Quebec Traumatic System.^4^

Experts highlighted that appropriate therapy in the pre-hospital field is very important. Many studies perform on the quality of pre-hospital services. Zafarghandi and Moeini argued that building a venous way and immobilization interventions were done before transporting patients to the hospital in 17.5% and 6.5% of studied cases, respectively; while no sufficient attention was paid to their airways.^30^ Assessment of prehospital management in a Thai university hospital showed patient management was appropriate in 80.5% of cases and inappropriate prehospital management was found in patients who presented with out-of-hospital cardiac arrest, and with chest pain.^31^

Based on the experts’ perspective, the designed indicators should be founded on clinical protocols in the hospital. The studies have reported the impact of protocols on mortality rates. For example, the results of Shakford et al. study in Sent Diego state showed that 7.6% of in-hospital mortalities in non-trauma facilities and 2% of them in trauma centers could have been prevented. Wrong diagnosis in a trauma hospital and, technical errors, side effects, and protocol violation in non-trauma hospitals were the causes of preventable deaths.^32^

Hospital indicators were the most mentioned indicators in this study. Patients’ disposition was one of these indicators. Delay in patients’ disposition will induce negative effects on patients care services according to the experts’ viewpoint. The studies confirm the validity of the experts’ views. Wills et al. indicated that three indicators were accompanied by the rise of the mortality rate. These indicators were abdominal surgery after 24 hours of patients’ entrance to the hospital, treatment of blunt compound tibial after 8 hours of the entrance, and no-immobilization of femoral diaphyseal fracture.^33^ It seems that identifying the cause of the delay in service delivery can help reduce it. Steren et al. reported Medicaid patients and those patients at university hospitals were associated with higher emergency department length of stay significantly.^34^

The experts believed that trauma-patients mortality is one of the indicators that is routinely extracted. Mortality has been investigated in various studies. Skaga et al. studied patients’ mortality in three periods including before discharging from hospital, before discharging from somatic care, and within 30 days after injury. According to their results, 95.4% of mortality occurred within 30 days after injury.^35^ This study demonstrated that the processes ultimately lead to the outcome. Therefore, process indicators are very important. The process of care allows a more comprehensive depiction of the functions that lead to a good or poor outcome.^25^ A study in the United Kingdom estimated that about 40% of improvements in patient outcomes was related to change in process performance.^36^

Calculating injury severity and survival likelihood indicators that are used to investigate and compare trauma centers based on experts’ views. Although mortality is a simple indicator, preventable mortality or survival likelihood is a more beneficial indicator, and provides more improvement in trauma care services.^37^ Drake et al. study showed that the preventable/potentially preventable death rate for the pediatric group was 21.0%, and for the adult group was 37.2%.^38^ Mousazadeh et al. reported mortality in the two traffic injuries groups with high and medium risk was 25.63% and in the low-risk group was o.42. It was lower than mortality risk was predicted for both severe and moderate risk (more than 50%) and low risk (less than 5%) in reference studies.^27^

The experts held that there should be some indicators for the post-discharge phase to evaluate the functional status of patients suffering from disability. A considerable portion of severe trauma patients suffer from long-term disabilities. According to studies, the functional status of these patients is below the normal level of society within 1 year after injury.^39^ Martino et al. reported 49.2% of trauma patients had some degree of disability. They concluded that follow-up screening programs can help patients and doctors in defining specific therapeutic-rehabilitation operations.^40^ There are some measures to evaluate the post-discharge functional status of patients. GOS, for example, concentrates on functional and participation levels such as participating in social affairs, transmission, returning to work, making communications, and participating in welfare activities.^37^

The experts stated that trauma care services need some infrastructures, including physical space and facilities. The study of Mock et al. evaluated WHO guidelines for the minimum facilities required from trauma cares in Mexico, Vietnam, Ghana, and India. They concluded that the required equipment is almost adequate in these countries. However, there were some gaps too, especially regarding the shortage of airway-related equipment, chest tubes and trauma drugs, and long waiting time for receiving some equipment such as radiography and laparotomy.^41^ Data was the key element of evaluation based on this study. Curtis et al. highlighted that trauma registry data are under-utilized and their use to drive clinical improvement and system/process improvement is vital to trauma quality improvement in Australia and New Zealand.^42^

The experts asserted that the employed indicators themselves need to be monitored and evaluated. Santana et al. argued that indicators are compatible with the knowledge available at study time and they need to be upgraded periodically. Also, they suggested that experts’ opinion alone is not sufficient for developing indicators. Such indicators indemnity performance guarantee should be evaluated; otherwise, irrelevant or non-executable indicators may likely be developed. On the other hand, indicators should be developed around the axis of patients and impacts and should be compatible with local environments and, actually, with the level of activities.^10^

The experts believed that an evaluation should be comprehensive. Similar to this study, a study was conducted with the cooperation of the Iran Ministry of Health and Medical Education to establish a performance management system in the emergency center of Ziaeian hospital. The project considered six group indicators including patient satisfaction, human resource empowerment (motivation, training, and attracting qualified employees), development of emergency center-related sub-indicators (equipment, information), emergency center expenditures, and quality of services.^43^

This study demonstrated that the evaluation could be done by internal and external evaluators. According to studies, external evaluation affects health care services. Governments, experts, managers, and insurance organizations have to determine new plans for public accountability, transparency of quality improvement, and value creation.^44^ Self-assessments and the assessment of service providers concerning the quality of their services is a principle used by JCAH (Joint Commission on Accreditation of Healthcare Organizations). It provides the opportunity of being aware of performance.^45^ It was similar to job-evaluation that was demonstrated in this study.

The experts also pronounced that disobedience to the referral system and lack of trained employees, who can provide services to patients with multiple injuries, make it difficult to provide suitable services. These problems are confirmed in developing countries in studies. For example, Albania lacks adequate and defined trauma hospitals. This issue causes patients not to be directed to hospitals with qualified services. On the other hand, there are only a few trained employees for trauma care services and this country needs an advanced education system for trauma surgeries, emergency medicine techniques, or life support programs for trauma patients.^3^ Chiria et al. in their study suggested that surgeons should contribute to the diagnosis, resuscitation, and management of at least 50 patients annually to be able to acquire emergency care standards certificate. Moreover, all physicians, nurses, and technicians should be trained with a multi-disciplinary approach to provide suitable services to patients with multiple injuries.^46^

Sometimes, it is necessary to consider other problems rather than just in-hospital issues in trauma care improvement, which are extra-organizational and need cooperation based on experts view. Other factors contribute to the increase in the rate of mortality and negative impacts on patients in studies. These factors may include unsafe roads, the concentration of population in high-risk areas with no hospital, and events associated with war, fire, landslide, natural disasters, accidents, lack of rules and laws, and incorrect execution of rules.^47^

## Conclusion

This study is one of the few studies related to the field of trauma care evaluation in Iran that was designed and conducted due to the lack of specific indicators related to the Iran context. In this study, experts described indicators including pre-hospital, in-hospital and, post-hospital according to the Iran health system. These indicators were raised according to the information system, human and financial resources. Experts confirmed to improve performance, in addition to relying on evaluation results, it is necessary to provide equipment, physical space, trained personnel, information system, and data registry system. Also, they confirmed trauma is an extra-organizational problem and requires vast resources and experiences, as well as local, regional, and national commitment, and participation of all relevant organizations. These identified indicators and evaluation steps and suggestions can be used by health policymakers and managers in Iran and developing countries with similar status.

**Acknowledgments**

This study is a part of PhD thesis. The authors are thankful to Research Deputy of the Tabriz University of Medical Sciences, Iran for his financial support and all experts for their scientific collaboration in the study.
